# 

**Published:** 1896-01

**Authors:** 


### CONTENTS.

COMMUNICATIONS.

The Pathology of Inflammation .......... 1
Four Requisites in Development.......... 13
Address by the President, Dr. W. H. Todd, D.D.S.......... 19
Diagnosis of Lesions of the Heart Previous to Administering Anæsthetics.......... 22
Some Thoughts on the Combination of Amalgam and Gold.......... 26
Dental Education.......... 29
The Use of Compressed Air in Dentistry.......... 34
A Rare Case.......... 39
A Suggestion about Cocain.......... 40
The Necessity of and Methods for Better Pulp Protection in Filling Teeth.......... 41
A National Dental Library and Museum.......... 43
The Office and Eccentricities of the Dental Pulp.......... 44

SELECTIONS.

A Safeguard.......... 45
Tobacco Insomnia.......... 46
Children's Ailments and Household Preventitives.......... 47
Local Anæsthesia.......... 47

NOTICES.

Pacific Coast Dental Congress.......... 48

EDITORIAL.

The Dental Colleges.......... 48
The Dental Register.......... 49
Officers Elected at Odontographic Society.......... 50
Ohio State Dental Society.......... 51
Obituary.......... 52

